# Exploring the Transformational Role of Regular Nature-Based Adventure Activity Engagement in Mental Health and Long-Term Eudaimonic Well-Being

**DOI:** 10.3390/bs15040418

**Published:** 2025-03-25

**Authors:** Gill Pomfret, Manuel Sand, Carola May, Jelena Farkić

**Affiliations:** 1 College of Business, Technology & Engineering, Sheffield Business School, Sheffield Hallam University, City Campus, Sheffield S1 1WB, UK; 2 Faculty of Sport Management, University for Applied Management, 19, 91757 Treuchtlingen, Germany; manuel.sand@fham.de; 3 Department of Hospitality, Tourism & Event Management, IU International University of Applied Sciences, Campus Hamburg, Waterloohain 9, 22769 Hamburg, Germany; carola.may@iu.org; 4 Academy for Tourism, Breda University of Applied Sciences, Mgr. Hopmansstraat 2, 4817 JS Breda, The Netherlands; farkic.j@buas.nl

**Keywords:** outdoor adventure activities, mental health, eudaimonic well-being, transformation, hedonic well-being

## Abstract

This article investigates the transformative impacts of regular nature-based adventure activity engagement and its long-lasting effects on eudaimonic well-being (EWB), specifically mental health. Although extant research highlights a wide range of well-being and mental health benefits from participation in such pursuits, less is known about experienced outdoor adventure enthusiasts for whom adventure is a fundamental and transformational part of their lives. The study builds on an existing conceptual framework that synthesizes pertinent research concepts on nature-based activity engagement and subjective well-being benefits. It presents key findings from 40 semi-structured in-depth online interviews with respondents from the UK, Germany, and Serbia. Interview data were collated and analyzed using a thematic framework approach. The findings highlight the importance of outdoor adventure activity engagement for respondents’ mental and physical health and long-term well-being. Regular activity participation can be transformational in reducing feelings of ill-being and enhancing EWB. It can improve self-efficacy and identity development and promote the fulfilment of psychological needs, facilitated by key transformational catalyzers. Continually entering a liminal state, experiencing emotions, and overcoming challenges and risks during engagement are crucial to “successful” long-lasting transformation. Further research should continue to explore adventure’s transformational and EWB benefits to develop long-term data.

## 1. Introduction

Good mental health is a state of well-being that involves the absence of psychological conditions and disorders as well as strong coping and resilience skills. It fosters individuals’ ability to manage everyday stress and fulfil their potential (Winefield et al., 2012; World Health Organization, 2022). It is a poignant theme in contemporary, post-pandemic society where ‘the world is undergoing a transition of unprecedented scale’ (Neuhofer, 2025, p. 2), which has resulted in a decline in mental health for some. This is borne out in the statistics, which show that approximately 25% of people globally were diagnosed with depression and anxiety in 2020 and medical interventions directed at curbing and treating higher incidences of poor mental health are increasing (United Nations, 2022). Relatedly, more people are “taking charge” of their own mental health through regular nature-based adventure activity participation, whether they have a diagnosed health condition or not (Pomfret et al., 2024).

Traditional approaches to investigating the concept of adventure have explored the role of risk, participants having “deviant” personalities, and the notion of “conquering” nature (Houge Mackenzie & Brymer, 2018). Although such approaches provide useful insights into the psyche of adventurers, they do not fully consider the complex and wide-ranging mental health motives that drive participation and the subsequent benefits. Our study explores these themes by considering adventure ‘in a mental health context, moving beyond traditional perspectives to a more inclusive and comprehensive understanding’ (Brymer et al., 2024, p. 1). Such an approach considers the well-being outcomes of adventure activity participation (Allan et al., 2020; Brymer et al., 2024; Farkić et al., 2020; Gass, 2025; Houge Mackenzie et al., 2021). Central to this is the opportunity to experience deep personal transformation and improve well-being through the interplay between participants, the activity, and the natural environment. This transformation manifests as potent inner journeys that lead to self-actualization (Maslow, 1954), involving self-exploration and self-realization (Sheldon, 2020). It can happen quickly through peak experiences, when the senses sharpen and thoughts are clear, eliciting feelings of immense joy (Maslow, 1975). Often, these experiences occur in natural settings, initiating a rapid and abrupt transformation (Naor & Mayseless, 2020), known as a “quantum change,” which results in a dramatic change in people’s core attitudes, feelings, beliefs, and actions (Bien, 2004). Scholars examine transformation alongside hedonic well-being (HWB) and EWB, which are key concepts associated with meaningful, beneficial outdoor tourism and recreation experiences (Câmara et al., 2023). While HWB is concerned with short-term positive emotions and ‘happy endorphins’ (Knobloch et al., 2017, p. 655) enjoyed during and immediately after the activity, EWB is longer lasting, as it is about seeking out the “true self” and creating meaning in life, resulting from satisfying the psychological needs for autonomy, competence, and relatedness, known as self-determination theory (Deci & Ryan, 1985). There are palpable synergies between these concepts, and EWB closely intertwines with mental health and transformation.

Our study aims to explore the transformative impacts of regular nature-based adventure activity engagement and its long-lasting effects on EWB, specifically mental health. It contributes to extant research in the fields of mental health and adventure in several ways. Firstly, there is limited literature (e.g., Pomfret et al., 2024) that considers experienced and frequent adventure activity participants in both recreational and tourism settings. Furthermore, extant tourism and recreation research mostly focuses on transformative experiences over a short duration and, therefore, gathers HWB data (Rus et al., 2022). Scholars tend to neglect the long-term effects on EWB (Câmara et al., 2023). By contrast, our study adopts a biographical interview approach and investigates the lifespan of outdoor adventure enthusiasts. Secondly, this study builds on an existing conceptual framework (Pomfret et al., 2023) that seeks to explain the subjective well-being (SWB) research concepts associated with adventure activity participation. Notably, the systematic literature review that led to the development of this framework established that studies on *physical and mental balance* gained from adventure activity engagement feature less in the literature (Brymer et al., 2024). Accordingly, our study responds to calls for further investigation into the mental health motives and benefits of engaging in such pursuits. By developing this understanding, we seek to embed the conceptual framework more firmly within the adventure recreation and tourism literature. Thirdly, our research adopts a cross-cultural approach through interviews with experienced outdoor adventure enthusiasts from Germany, Serbia, and the UK. Scholars underutilize this approach in adventure research, even though adventure tourism and recreation are represented and perceived differently from one culture to another (Sulaiman & Wilson, 2018).

## 2. Literature Review

This literature review explores the extant research themes that are pertinent to the aim of this study. Firstly, we present the conceptual framework (Pomfret et al., 2023) that forms the foundation of this study and highlights the key well-being research concepts of nature-based adventure activity engagement. Secondly, we explore the psychological needs that facilitate well-being through self-determination theory (SDT; Deci & Ryan, 1985). Thirdly, we outline the transformation literature and the five key catalyzers of transformative tourism and recreation (Rus et al., 2022), applying these to the core elements of nature-based adventure. The review demonstrates the inextricable links between these concepts and the wide-ranging opportunities for transformation, improved mental health, and long-lasting EWB that outdoor adventure pursuits offer.

### 2.1. Subjective Well-Being (SWB) and Nature-Based Adventure Activity Engagement

**Figure 1:**
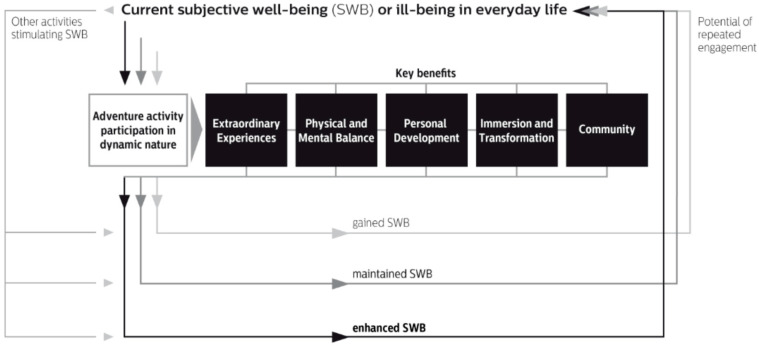
Conceptual framework (CF): Outdoor adventure activity participation and subjective well-being.

Figure 1 illustrates the conceptual framework (Pomfret et al., 2023) and synthesizes the research concepts pertinent to nature-based adventure activity engagement and SWB benefits. The framework comprises five metathemes, which are inextricably linked, and three different levels of SWB, namely, gained, maintained, and enhanced. Each metatheme has several subthemes, amounting to 16 in total. Activity participants ‘will enjoy different levels of SWB if they experience one or more subthemes within one or more of the five metathemes during and/or at the end of their activity’ (p. 4). Regular outdoor activity engagement, at home and/or on holiday, can facilitate increasing levels of SWB.

Although the framework comprises five interrelated metathemes, the *physical and mental balance* metatheme aligns best with this current study as it focuses on mental health as a core element of long-term EWB. Even its *physical health* subtheme, which features in many adventure-related studies (e.g., Brymer & Feletti, 2020; Doran, 2016; Farkić & Taylor, 2019), is strongly linked with mental health. For instance, increased fitness leads to improved health and vitality, and physically challenging one’s body promotes lifestyle changes and strengthens individuals’ desire to be active, all of which facilitate enhanced mental health. Extant research on the subtheme *mental health and emotional balance* has ascertained that adventure participants enjoy mental health benefits such as psychological restoration (White et al., 2013), alleviation of stress (Pomfret et al., 2024), reduced anxiety and feelings of hopelessness (Davidson & Ewert, 2024), faster recovery from setbacks in life (Gstaettner et al., 2018), increased mental energy, emotional balance (Buckley & Westaway, 2020), improved self-efficacy and self-confidence (Ewert & Davidson, 2021), flourishing, and a more positive and relaxed lifestyle (Houge Mackenzie & Brymer, 2018). Research also indicates that regular engagement in outdoor adventure activities can foster long-term EWB. These participants enjoy more enhanced mental health benefits than those who only casually or occasionally dip into these pursuits (Wolf et al., 2014). Notably, scholars have primarily examined the benefits women gain from regular adventure activity engagement, whereas our study explores the perspectives of different genders. Women who trail run frequently enjoy being part of a running community, improve their self-confidence, feel more able to embrace and reframe change better, and experience improvements in their mental health (Lincoln, 2021). Women who frequently hiked together developed resilience, as this activity ‘had rescued them psychologically from dark and difficult times’ (Buckley & Westaway, 2020, p. 6). Pomfret et al. (2024) ascertained that regular outdoor activity participation in national parks improved people’s mental health and positively impacted coping, resilience, and stress alleviation. Individuals are increasingly seeking out these benefits to counterbalance their often fast-paced and frenetic lifestyles (Pomfret et al., 2024) and to give their lives purpose and meaning (Alizadeh & Filep, 2023).

*Challenge and risk* and *coping and resilience* are two other subthemes within this metatheme that are concerned with mental health. Adventure pursuits facilitate resilience-building and coping skills in everyday life as such activities often involve dealing with risks and challenges and developing learning strategies to deal with demanding situations (Buckley & Westaway, 2020; Ewert & Davidson, 2021). Engaging in these pursuits, therefore, offers a plethora of opportunities that are often unavailable in everyday life (Reid & Kampman, 2020). Participation also fosters self-reflection, improved self-concept and self-esteem, and enhanced EWB (Fletcher & Prince, 2017). Despite these rich insights, which confirm the well-being outcomes of nature-based adventure activity participation, the role of these pursuits in fostering EWB has not yet been fully explored (Houge Mackenzie & Hodge, 2020). Relatedly, there is also a lack of interdisciplinary research on the long-term well-being effects of tourism on mental health (Chen et al., 2024). This may be because tourism providers tend to focus their efforts on offering hedonic pleasure, neglecting the longer-term benefits of holidays (Aldossary & McLean, 2022). Although our study not only explores adventure tourists but also adventure recreationists, such research gaps signal a clear need for further work in this area. Many practitioners already use adventure activities as mainstream therapy interventions, palpably recognizing the benefits of such experiences for mental health (Clough et al., 2016). However, there is a lack of ‘evidence-based knowledge’ (Britton et al., 2020, p. 52), which would be useful in developing policy on the therapeutic effects of nature-based adventure activities, providing support for implementation in struggling healthcare systems, e.g., new and innovative social prescribing programs to improve public health (Raymond et al., 2017). Accordingly, although we know something about the strong association between mental health and nature-based adventure activities, more research needs to be performed in this area. This current study, therefore, attempts to address this knowledge gap, given that improving mental health in contemporary society is a prominent concern.

A second metatheme that is clearly aligned with this study is *immersion and transformation.* Extant research on transformation through leisure experiences has primarily focused on tourism settings (Rus et al., 2022) and includes types of transformative tourism (Câmara et al., 2023), the design of transformative experiences (Neuhofer, 2025; Sheldon, 2020), catalysts and constraints of transformation (Pung & Del Chiappa, 2020), a transformative travel experience scale (Soulard et al., 2020), product development (Santos et al., 2020), and how nature-based transformative tourism can facilitate sustainability at the destination level (Seocanac, 2022). The transformational benefits of volunteer tourism, backpacking, and study tours are particularly well-researched because such experiences offer extensive opportunities for personal growth (Anderson et al., 2013; Blocker & Barrios, 2015). For instance, volunteer tourists often seek meaningful, authentic experiences and the opportunity to build strong relationships with the host community (Magrizos et al., 2021). There is limited research on the transformation process (e.g., Tasci & Godovykh, 2021) to understand how transformative experiences can positively influence participants’ well-being (Huang et al., 2024). Accordingly, our research explores this transformation process relative to adventure settings and experiences and the EWB benefits (focusing on mental health) from activity engagement.

Adventure experiences have strong potential to offer immersion and transformation benefits, as they encourage individuals to disconnect from their everyday lives through *antistructural experiences/liminality* (Turner, 1974). Liminality involves venturing from the mundane to the extraordinary (i.e., the adventure setting) for individuals to enjoy the freedom to be themselves (Muldoon, 2020; Rus et al., 2022), then returning to everyday life feeling transformed to a greater or lesser extent. As participants become fully absorbed in the activity and the natural environment, they potentially experience stronger liminality (Goodnow & Bordoloi, 2017; Houge Mackenzie & Goodnow, 2021). This interaction with nature during activity engagement is represented in the *human-nature* subtheme, whereby individuals employ all their senses to feel “at one” with nature. The natural environment heightens our senses and provides a backdrop for enriching and transformative experiences, resulting in feelings of awe and improved well-being (Folmer et al., 2019). Being immersed in nature offers immeasurable opportunities for people to re-set and enhance their mental health (Buckley & Cooper, 2022). Nature offers a liminal space to enjoy extraordinary experiences and the opportunity to reach new states of being (Conti & Cassel, 2020). Deeply immersing oneself into nature fosters a sense of belonging and embodiment, transcendental and spiritual experiences filled with awe, a feeling of belonging to something bigger, and the development of eco-centric viewpoints (Cohen et al., 2010; Wyles et al., 2016). Relatedly, the *rhythm of nature and resonance* subtheme involves synchronizing one’s own rhythms with those in nature. For instance, sea kayakers adopted a diurnal rhythm and followed the lunar cycle during their journey around a tidal island (Varley & Semple, 2015). The *deceleration and mindfulness* subtheme concerns slowing down and encountering mindfulness, sometimes during challenging adventure activities (Mykletun & Mazza, 2016).

There are three more metathemes in this conceptual framework that merit attention as the boundaries between each metatheme are blurred, and we propose that each one influences individuals’ mental health and EWB in its own way. The metatheme *extraordinary experiences* comprises three subthemes. The first two are *natural highs* and transcendental *experiences and awe*, and there is plentiful research on these subthemes (Gstaettner et al., 2018; Reid & Kampman, 2020). However, the subtheme *optimal flow and peak experiences* is most researched (e.g., Buckley, 2012; Farkić et al., 2020). This is most likely because flow (Csikszentmihalyi, 1975) and peak experiences (Maslow, 1954) are pioneering, well established, and extensively researched psychological constructs that are strongly associated with EWB. Peak experiences are emotionally charged moments of extreme happiness through novel and spontaneous experiences that encourage nature immersion and personal development (Maslow, 1959). *Personal development* is the most researched metatheme, which is unsurprising given the challenges and unpredictable situations that characterize many adventure activities, and the growth that participants experience through successfully dealing with these. Researchers (e.g., Ewert et al., 2020; Farkić et al., 2020) have explored the subthemes of *learning and knowledge acquisition* and *flourishing*. However, the subtheme *individual identity* is of most interest to adventure scholars, reflecting ‘who you are or equally who you are not and also who you would like to be’ (Myers, 2010, p. 118). When participating in adventure activities, individuals can become different people, reinventing themselves through taking on new challenges, pushing themselves out of their comfort zone, and enjoying novel experiences (Ashworth, 2017; Doran et al., 2020). The final metatheme is *community*, which reflects how adventure experiences can create a sense of “we” (Simpson et al., 2014). The subthemes are *collective identity*, which manifests itself through the many and varied opportunities for camaraderie during adventure activity engagement (e.g., Allman et al., 2009), and *beneficence*, which is simply defined as ‘acting in ways that benefit others’ (Houge Mackenzie & Hodge, 2020, p. 31).

### 2.2. Self-Determination Theory, Transformation, and Well-Being

Self-determination theory (SDT), a psychological construct proposed by Deci and Ryan (1985), is often applied to tourism and recreation settings (e.g., Câmara et al., 2023; Lee & Ewert, 2019) to explain transformative experiences. The theory is relevant to this current study because it is based on the premise that fulfilment of three core psychological needs can lead to transformation and EWB. These needs are ‘autonomy (i.e., one’s eagerness for psychological liberty and freedom of will), competence (i.e., one’s desire to control outcomes and experience mastery), and relatedness (i.e., one’s need for closeness and connection with others)’ (Huang et al., 2024, p. 976). If individuals cannot fulfil these needs during their everyday lives, then ill-being may result, which is characterized by negative affect, e.g., unpleasant moods and emotions. Such feelings impact positive psychological functioning and can lead to reduced mental health (Diener, 2006). Houge Mackenzie and Hodge (2020) proposed that individuals fulfil SDT needs through adventure activity participation, resulting in optimal levels of EWB and enhanced mental health. Frequent climbers enjoyed higher levels of autonomy, relatedness, and competence in their everyday lives compared with a control group (MacGregor et al., 2014). This is because climbing regularly ‘provides participants with an agentic emotional experience that then benefits their everyday functioning; such benefits are not derived from other (low-risk) activities’ (p. 175). Adventure programs, education, and recreational activities, which specifically target the three SDT needs (Lloyd & Little, 2010; Tsaur et al., 2015), also enhance EWB. The greater the satisfaction of needs, the stronger the psychological well-being markers, such as meaning, purpose, significance, and EWB. Although these findings confirm the inextricable links between adventure, SDT needs fulfilment, and EWB, there are other ways to achieve and sustain long-term EWB.

Beneficence is a more recently recognized psychological need, which extends SDT and fosters long-term EWB. It is integral to Houge Mackenzie and Hodge’s (2020) conceptual framework, which illustrates how adventure experiences can enhance EWB through needs satisfaction and contact with nature. Beneficence manifests itself through partaking in activities that foster prosocial behavior, environmental awareness, and stewardship. These behaviors strengthen individuals’ desire to protect nature (Huynh & Torquati, 2019; Wolf et al., 2014). Beneficence can lead to peak experiences where individuals enjoy self-transcendence and the highest level of human consciousness (Maslow, 1975). Building on SDT theory, Huang et al.’s (2024) study on transformative tourism experiences found that the fulfilment of six psychological needs is important for tourists’ well-being. These are *escapism, relatedness, aesthetics and relaxation, education, beneficence, and self-challenge and competence.* They developed an ‘activities-needs-well-being’ (p. 974) conceptual framework to highlight the influence of transformative tourism on individuals’ well-being through satisfying these needs. Such frameworks can encourage more ‘health and well-being approaches to adventure recreation’ (Houge Mackenzie & Hodge, 2020, p. 34) and, in turn, influence the perspectives of public policymakers and practitioners who offer adventure experiences.

### 2.3. Transformative Tourism and Recreation Catalyzers

Rus et al. (2022) proposed five key catalyzers of transformative tourism, which clearly align with the core elements of nature-based adventure outlined in our conceptual framework (Pomfret et al., 2023). Firstly, *risk, challenge, and novelty* are integral to original (e.g., Ewert, 1985) and contemporary definitions (e.g., Pomfret et al., 2023) of outdoor adventure. This set of catalyzers facilitates positive attitudinal and behavioral changes and helps individuals to manage everyday challenges (Folmer et al., 2019). In an adventure context, German tourists reported spending more time outdoors post-holiday, were keener to adopt a healthier, more sustainable lifestyle, and developed a deeper appreciation of other cultures (Gross et al., 2023), indicating positive behavior change. Secondly, *authenticity and liminality* concern existential authenticity, which is a state of “being” (Wang, 1999) facilitated by meaningful, profound, self-fulfilling, and optimal experiences (Câmara et al., 2023; Kirillova et al., 2017), such as those offered in adventure settings. Developing new knowledge, skills, and beliefs also encourages existential authenticity, leading to transformation and positive effects on long-term behavior change (Pung & Del Chiappa, 2020). The search for authenticity motivates individuals to escape their usual everyday lives and immerse themselves in a different environment where they can experience liminality (Turner, 1974).

The third set of catalyzers, *social dynamics and communitas*, facilitates connections with others, tolerance, humility, kindness, and a broader mindset in tourism settings (Brooks, 2020). Participants develop communitas during adventure engagement, as activities involve accepting new challenges and responsibilities, pushing personal boundaries, and ‘reinventing themselves’ (Ashworth, 2017, p. 220). *Emotions and peak experiences* are the fourth set of catalyzers, which are coupled together because emotions are often precursors to transformation through peak experiences (Pastor & Kent, 2020). Emotions are integral to adventure experiences and, depending on the activity, participants can experience emotional peaks and troughs (Pomfret, 2012) such as ‘terror and elation, joy and despair, [and] anxiety and pleasure’ (Swarbrooke et al., 2003, p. 14). *Self-reflection* is the fifth catalyzer and a critical part of the transformation process, as it encourages individuals to attribute meaning to their trip and, subsequently, enjoy long-lasting well-being benefits (Pung & Del Chiappa, 2020). In an adventure context, prominent studies, such as Arnould and Price’s (1993) seminal work on the meaning of white-water rafting for participants, highlighted the importance of self-reflection. Unaccustomed to rafting, participants enjoyed intensely emotional and hedonic “extraordinary experiences,” enhanced by communing with nature and its rejuvenating effects, connecting to others, communitas, personal development, and renewal of self. These transformative experiences were conceptualized as “river magic” because of the remarkable settings in which the rafting activities occurred. A summary of the key theoretical concepts informing this study, and discussed above, is presented in Table 1.

## 3. Materials and Methods

To investigate the research aim—to explore the transformative impacts of regular nature-based adventure activity engagement and its long-lasting effects on eudaimonic well-being (EWB), specifically mental health—we adopt qualitative values and examine the key research themes ‘as if through the eyes of the people being researched’ (Matthews & Ross, 2010, p. 28), with the aim of understanding people’s feelings and experiences (Neuman, 2006). We carried out 40 online qualitative, in-depth semi-structured interviews across three countries: Germany, Serbia, and the UK. Based on a purposive sampling approach, we invited individuals to participate in interviews from our network of personal contacts and from within outdoor adventure activity communities, e.g., clubs and associations, in the respective countries. We selected individuals who had been actively participating in nature-based adventure pursuits for a minimum of three years or longer. Such activities included hiking, climbing, canoeing, caving, mountain biking, forest bathing, bushcraft skills, and snow sports. Respondents engaged in these, either independently or through an outdoor activity operator, and/or belonged to an activity club or a society. Being members of the Adventure Tourism Research Association, academic researchers in adventure and outdoor sports, experienced adventurers, and sport club members ourselves enabled us to contact respondents and invite them to participate in the research project. Being aware of our own bias due to our academic background and personal interest in adventure, we opted for a reflexive approach. This helped us to remain aware of our personal perspectives throughout the interviews and to work toward a more nuanced interpretation of the data, enriching the analysis through double hermeneutics (Nizza et al., 2021). This concept refers to the idea that social scientists interpret and analyze the meanings created by individuals in society, while these individuals, in turn, can adopt and be influenced by the interpretations and theories developed by social scientists. While this research considers key metathemes from the extant systematic literature review (Pomfret et al., 2023) for our outdoor adventure enthusiasts, we did not set out to “test” the relevance of these or any preconceived notions we had developed from this initial study’s findings. We did not formulate any specific theories or derive hypotheses that were subsequently tested. Our aim was to determine the significance of the individual metathemes for the interviewees. Therefore, our approach to interview data collection was inductive, but with an exploration of these metathemes.

### 3.1. Sample 

To gather a varied sample, respondents were drawn from diverse backgrounds, age groups, and regions of each country. The sample included 21 male and 19 female respondents and people aged 18–64 years old, as indicated in Table 2. Although the table highlights that respondents within this study spanned a broad range of ages, the majority were aged 35 years plus. This skew toward older age groups reflects the requirement that respondents needed to be experienced outdoor enthusiasts, and adventure activity participation can be costly and time consuming (Adventure Travel Trade Association [ATTA], 2020).

The sample allowed us to investigate our research questions with outdoor adventure enthusiasts. With their extensive experience in the field, they were able to answer the questions retrospectively and to reflect on the transformational aspects of their adventures. 

### 3.2. Interviews

The interviews took place between October 2023 and May 2024 and lasted between 30 min and one hour and thirty minutes. The mean interview duration was 58.5 min, with a standard deviation of 2.6. The interviews were conducted online via Zoom or Teams in the respondents’ native languages, and transcripts were generated. The transcripts were anonymized, and respondents were allocated a pseudonym to anonymize the reporting of findings. The research team translated the German and Serbian transcripts into English. The interviews were carried out by all four researchers, who encouraged respondents to be open and honest in their responses. Icebreaker questions explored the respondents’ favorite free time activities, how and why they started adventure activities, and what came to their mind when they thought about the word “adventure.” The interviews were recorded and transcribed, with a copy of the transcript sent to each interviewee for checking and reflection. The questions were open, and we invited respondents to talk about their opinions and experiences, sometimes probing with follow-up questions to further examine a topic. We gave respondents as much time as they needed to fully answer the questions so that they did not feel rushed. As we adopted a semi-structured approach to the interviews, respondents often answered later questions earlier on. Therefore, we skipped the questions they had already responded to, sometimes returning to these later to gather more detailed responses.

### 3.3. Interview Themes and Questions

The questions were designed to probe respondents about the 5 key themes and 16 subthemes derived from the conceptual framework (Pomfret et al., 2023) presented earlier. Following the icebreaker questions, we asked how respondents got involved in adventure activities. The interview schedule included three questions on the theme of *extraordinary experiences* (e.g., “Have you ever had any outdoor activity experiences which stick in your mind and that still trigger intense emotions when you think of them?”) and four questions on *physical and mental balance* (e.g., “What role do activities play in terms of your physical health and well-being?”). This was followed by four questions on the theme of *personal development* (e.g., “What have you learned about yourself through doing these activities?”), three questions on the theme of *immersion and transformation* (e.g., “Are there times when you feel you are merging with nature when you are doing your activities? If so, how does that feel?”), and four questions on the theme of *community* (e.g., “How important is being part of a group (or being loosely connected to a group) when doing your activities? What benefits does this bring?”). At the end of the interview, we asked three general questions designed to elicit summaries of the themes that were most significant in their adventure journeys. The interview schedule also included follow-up and more probing questions.

### 3.4. Data Analysis

Once data collection was complete, the transcripts (including the transcripts translated into English) were checked thoroughly against the video recordings. Following this, we used a thematic framework approach to analyze the data (Ritchie & Spencer, 1994). For this paper, the focus was on respondents’ outdoor adventure activity experiences, their transformative effects, and how these influence their mental health and well-being. After the initial “familiarization” stage, which involved reading the transcripts and listening to the recordings, we developed a “thematic framework” to refine the data. We then marked phrases and aspects important to our research aim (“indexing”). Next, “charting” the data involved designing charts for each key theme and inputting the relevant data. In the final “mapping and interpretation” stage, we revisited the research questions; reviewed the framework, indexes, and charts; examined different sections of the transcripts for similarities, differences, and explanations; searched for connections between the data; and reflected on the relevant themes.

### 3.5. Trustworthiness

We used Tracy’s (2010) criteria for thorough qualitative research to ensure the trustworthiness of this study. We met at each stage of the data analysis process to review the findings and our assumptions about these, and to discuss the emerging themes. This allowed for a general understanding of the data and helped to minimize subjective assessment of these. We interpreted respondents’ experiences from their perspectives, followed by re-interpretation of those insights within the broader theoretical and positional understanding of the topics. Our own backgrounds shaped how we understood and interpreted respondents’ realities. As ‘good qualitative research delves beneath the surface to explore issues that are assumed, implicit, and have become part of participants’ common sense’, this was an important methodological procedure (Tracy, 2010, p. 843). When reporting the key findings, we introduce some lengthy quotes from respondents to illustrate the rich descriptive accounts of their adventure experiences, and to enhance credibility of the findings for readers. This comprehensive data analysis process is a type of triangulation (Ritchie & Spencer, 1994) that makes use of the whole research team to gather different researcher viewpoints and to deepen the understanding of the key findings. 

## 4. Results and Discussion

This part of the paper outlines the key interview findings related to the study’s aim: to explore the transformative impacts of nature-based adventure activity engagement and its long-lasting effects on EWB, specifically mental health. We present findings that reflect the framework’s (Pomfret et al., 2023) *physical and mental balance* and *immersion and transformation* metathemes. We also briefly touch on the three other metathemes (*extraordinary experiences, personal development, and community*) to acknowledge their intertwined nature. Relatedly, we introduce aspects of SDT (Deci & Ryan, 1985) and extant research in tourism (Huang et al., 2024) and outdoor adventure settings (Houge Mackenzie & Hodge, 2020) to illustrate the importance of fulfiling fundamental psychological needs (*autonomy, competence, relatedness, beneficence, escapism, aesthetics and relaxation, education, and self-challenge*) for good mental health and EWB. Furthermore, we apply pertinent transformation catalyzers (Rus et al., 2022) to explain the findings (*risk, challenge and novelty, authenticity and liminality, social dynamics and communitas, emotions and peak experiences, and self-reflection*).

### 4.1. The Importance of Outdoor Adventure Activity Engagement to Mental and Physical Health

Although the findings presented in this paper focus on mental health, we recognize that outdoor activity engagement is important to physical health and that this is inextricably linked with mental health. Respondents’ comments suggested that participation facilitated their physical fitness, endurance, stamina, and strength; improved energy levels; provided opportunities to test their physical limits; and enhanced their physical health. Physical exhaustion was often viewed positively, as it encouraged respondents to detach themselves from their everyday environment. These findings align with previous research (Brymer & Feletti, 2020; Gstaettner et al., 2018) that suggested that physically challenging the body is beneficial to physical as well as mental well-being. This is exemplified in Sabine’s comments:

‘This [outdoor activity] naturally creates a sense of well-being. When you have moved in wind and weather, you appreciate being inside again. And, of course, exercise does something to you. You gain muscles, you gain stamina, you gain inner and outer strength. So, you’re less vulnerable and your immune system is strengthened.’

Respondents’ narratives illustrated that regular engagement in nature-based adventure activities fostered good mental health, emotional balance, and long-lasting EWB. Many alluded to, or openly stated that, the “great outdoors” was critical to their mental health. Tony’s words that ‘not being able to do outdoor activities would almost be like not being able to breathe’ highlight this well. Rich descriptions of their activities often noted that these experiences provided opportunities for sustaining their mental health that were not easily accessible in their everyday lives. This aligns with extant research (Reid & Kampman, 2020, p. 1) that noted that ‘going into the unknown’ with its associated challenges and uncertainties facilitates improved mental health more than doing everyday routines.

Several respondents talked about “grounding” to describe the importance of adventure activities to their long-lasting mental health. This is reflected in Paul’s comments:

‘My mental health is so much better if I can get out on the hills and see those wide spaces and feel that wind on my face and, you know, have those experiences … If I feel in trouble emotionally, then I’ll try and get grounded. With every aspect of my life, I try to do that. But I learned it in the mountains. … And if I have a long-extended period of not being in the mountains, my mental state just gets worse and worse, you know.’

Paul’s words signal the critical role that the natural environment plays in their mental health, corresponding with extant research (Buckley & Cooper, 2022). Others noted that they regularly immersed themselves in outdoor activities because they provided ‘a form of therapy’ that ‘makes me feel good again’ (Tanja).

### 4.2. Ill-Being, Well-Being, and Liminality

Although well-being is currently a prominent theme within adventure research (e.g., Brymer et al., 2024), it is also important to consider the potential of outdoor adventure activities for reducing ill-being (Pomfret et al., 2024). Contrary to the misconception that ill-being is the direct opposite of well-being, whilst it concerns the absence of well-being, it additionally involves the negative outcomes of a bad experience, often manifested as anxiety or depression (Ryff et al., 2006). Furthermore, the absence of ill-being does not necessarily infer high levels of well-being (Keyes, 2002). For some respondents, regular activity engagement facilitated their recovery from mental health conditions, such as depression and post-traumatic stress disorder (PTSD). For instance, for Susan, who suffered with PTSD, outdoor activities were ‘really really good at levelling me emotionally,’ helped to ‘evaluate my emotions’, and ‘give me that balance back’. Such pursuits encouraged them to feel like they were ‘succeeding rather than failing, which post-traumatic stress makes you feel’. These findings align with the work of other scholars that highlights how nature-based activity engagement can expedite recovery from life’s problems and challenges (Gstaettner et al., 2018). They also signal that the process of recovery from mental health issues, for some, is contingent on frequent and long-term engagement with transformative adventure experiences, resulting in sustained EWB. In the words of Miroslav, adventure experiences are driven by the need to complete a ‘mental task’ which ‘releases accumulated stress, redirects thoughts and helps in maintaining mental hygiene’. The resultant effect is that participants often reflect on their adventure experiences, making them feel more emotionally balanced (Pomfret et al., 2023). Psychological needs such as *relaxation* and *escapism* are fulfilled (Huang et al., 2024), and the transformative catalyzers of *authenticity and liminality* are triggered (Rus et al., 2022).

Many respondents recounted intermittent periods of stress and anxiety in their lives, which prompted their frequent participation in adventure pursuits, albeit these were not the only reasons encouraging engagement. They “exploited” outdoor activities to counteract any feelings of negative affect they experienced pre-engagement and to build their levels of well-being. They often returned to their everyday lives post-activity with enhanced HWB. This is illustrated in Tobias’s comments, who explained that running facilitated a meditative state, where they no longer thought about anything:

‘I do it [running] when I’m stressed and when something is really getting on my nerves. After an hour, I come home “refreshed” and have actually run off the stress and sorted my thoughts. And that’s also possible when hiking and [generally] through physical exercise. … Somehow, I can also purify myself a little on the inside.’

This temporary state of HWB transcended to long-lasting EWB through regular adventure activity participation over a lengthy duration. This is apparent in their comments that show how they could readily overcome difficult situations and rely on their own abilities to make the right decisions in everyday life. Facing challenges during outdoor activity participation helped them to handle stress in a positive way, fostered decision making, and promoted a sense of emotional balance. Melanie’s comments highlight this point:

‘You become more stress-resistant overall. So, if I go hiking at the weekend and then work on Monday, Monday always brings a certain stress factor with it. The relaxation lasts longer after such a hiking weekend. I think that simply makes me more relaxed as a person, perhaps also towards other people and stressful situations.’

Notably, the type of activity in which respondents participated also affected whether they experienced HWB, EWB, or both types of well-being. Activities that were longer lasting tended to generate intense memories and a sense of accomplishment and EWB. For instance, Anja reported that ‘after longer outdoor activities, I feel happy for weeks and radiate positive energy, which also carries over into my everyday life.’ Similarly, Nigel alluded to a stronger, enduring sense of well-being from their more meaningful and cherished Iron Man experience:

‘So, I came back from a climbing trip in France and then got on this problem, Nemesis, which was a 7C. I could remember absolutely everything about it. And I did it. It just came out of nowhere. So, it was a really good sort of feeling, like “Oh, my God, I’ve done that.” But that was really short lived, that sort of feeling. Of accomplishing that. Whereas the accomplishment of the Iron Man, that feeling, that’s still there. It’s still there. It’s still quite strong. You know that sense of personal achievement.’

It is difficult to say when and where respondents “got hooked” on adventure, although school, friends and family, or clubs (e.g., scouts) facilitated their initial engagement in outdoor pursuits. It is notable that the continuity and frequency of participation exert a long-lasting effect on mental health and EWB (Pomfret et al., 2024). Respondents reflected on periods of their lives when they could not partake in outdoor activities, indicating strong feelings of ill-being and the absolute necessity for adventure experiences to maintain and enhance their EWB. Tiffany’s recollection of how they felt during the pandemic’s outdoor restrictions emphasized these feelings of ill-being: ‘I probably felt a bit depressed, and I didn’t feel as if I coped as well. I think it shows that being outdoors for me is really important.’ Observations from others also exemplified negative affect during this era. For instance, Frank noted that ‘there was a fire burning inside me and I was dissatisfied … I’d be heartbroken if I couldn’t do it [diving] anymore.’ Kimberly talked about a time when they had injured their knee and how they became depressed after a few weeks ‘even though nothing had objectively changed’ and they thought they had a ‘positive outlook.’ Yet, they thought they would ‘use this time to read or do some sedentary hobbies. But I just became miserable pretty quickly.’

Accordingly, immersion in their activities within nature helped to combat ill-being, temporarily “removing” respondents from their usual environment to one in which they could escape their worries and be themselves (Houge Mackenzie & Hodge, 2020). Being outdoors offered a liminal place where respondents could be who they want to be, feel more relaxed, and become more creative (Pomfret et al., 2023). For instance, Kate considered themselves to be ‘a massive massive overthinker’ and a ‘massive worrier’, yet when they are hiking, they ‘don’t have to think about things’ or ‘worry about anything’, which gives them a sense of release and moments of inspiration when ‘ideas just come to me’. By immersing themselves into this liminal space during activity engagement, Thomas explained that ‘I can’t stand out socially in Zoom meetings or feel as great as I do when I’m laughing and sailing or laughing and hiking. That just doesn’t work.’ Therefore, having this safe space gave them a feeling of self-worth and influenced their identity formation. Mary reported that ‘at a time in my life when I didn’t have much confidence, I think being able to do adventurous things and build an identity around adventurous things gave me more confidence.’ The natural environment played a critical role in reaching this state of liminality, and respondents alluded to how attuned they were to this setting, as noted by Sabine:

‘I perceive nature differently than people who aren’t outside as much. I know when the leaves fall in autumn, when and where mushrooms grow, and I notice how dry it is, whether it’s drier than the previous year … I perceive the animals differently, and I notice even the smallest changes because I’m out there so often. I’m outside because I truly value nature and am mindful of what it does for us, and especially what we are doing to it.’

To summarize, these findings illustrate that outdoor adventure activity engagement has strong potential to reduce feelings of ill-being and enhance HWB and EWB. Being active in nature can have a therapeutic effect on respondents who are facing mental illness, and it can prevent such issues for those who have good mental health. Liminality and frequent participation over time palpably act as mediators for sustained well-being.

### 4.3. Self-Efficacy and Identity Development

Respondents alluded to how outdoor adventure improved their self-efficacy, which refers to ‘beliefs in one’s capabilities to organise and execute the course of action required to produce a given attainment’ (Bandura, 1997, p. 3). People with high self-efficacy have an optimistic view on life and the right “resources” to deal with demanding situations, which leads to enhanced well-being (Paciello et al., 2016). In an adventure context, self-efficacy promotes outcomes such as hardiness, creativity, and social skill development (Ewert & Davidson, 2021). High levels of self-efficacy are often necessary to successfully overcome obstacles when participating in challenging adventure activities that involve uncertainty and potential risks (Doran & Pomfret, 2019). However, being in control and not pushing oneself excessively can also lead to improved self-efficacy. For instance, when recounting different hiking experiences, Annabelle expressed that:

‘I think it’s good for me to do that [solo hiking] and to recognise it ’cause I don’t give myself a lot of credit, just generally, and my partner’s very good at making sure I recognize that as well. And just, I think it is important for me to feel capable or like I’ve achieved something, and I think the outdoors is a really good vehicle for that and it’s why everything I’ve said about staying in my comfort zone is important.’

Annabelle suggested that they relied on their solo outdoor achievements to continually foster self-efficacy, hinting that they could not accomplish this in their everyday life. Reflecting previous work (Doran, 2016), respondents commented on the sense of empowerment they gained from completing very demanding activities, and mentally, ‘how incredibly good they are for me’ (Patrick). One respondent noted that ‘once you have conquered a high pass [in hiking] or something difficult, and have surpassed yourself, even if you are scared, then you definitely gain mental strength’. Therefore, if respondents exceeded their original expectations for a particular activity, their self-efficacy could increase exponentially. This may help to explain why respondents were outdoor adventure enthusiasts, as activity engagement offers wide-ranging opportunities to experience liminality and their transformation over time. Or, as Dieter put it, ‘you don’t come out of a forest the same way you went in, and that’s how it is for me.’ The process of activity participation therefore raises self-efficacy and transforms individuals. Related to self-efficacy and an improved sense of self, respondents talked about how adventure activity engagement transformed who they are, that is, their individual identity, aligning with extant research (Ashworth, 2017; Myers, 2010). For women in particular, adventure settings offer feelings of freedom, personal development, and self-confidence, resulting in an “adventure identity” (Doran et al., 2020). As Milena recounted ‘whether they [adventure activities] have changed me as a person, I don’t know. But they make up a part of who I am. That is certain.’ Often, identity development through adventure was a “turning point” in respondents’ youth, as illustrated by Mary: ‘I think being able to do adventurous things and build an identity around adventurous things gave me more confidence at the time.’

A key benefit of strong self-efficacy is the ability to trust oneself to manage everyday challenges (Ewert & Davidson, 2021), as Steve commented:

‘In so far as the perceptions of your own potential and just your efficacy as an individual and because I’ve faced some, you know, serious stuff in the outdoors. I’ve overcome them. So, you know, you can. So essentially, I think I’ve got more sort of self-belief in my abilities to overcome most things because most things can be dealt with.’

Although respondents thrived from relying on their own skills and abilities to successfully navigate their adventure journeys, and sometimes “adventuring” alone, they also emphasized the importance of belonging to outdoor communities. These positively impacted self-efficacy and identity development, as respondents shared experiences with people who had the same interests. Outdoor communities also fulfil relatedness needs (Houge Mackenzie & Hodge, 2020), reflect *social dynamics and communitas* (Rus et al., 2022), facilitate identity formation, and develop self-confidence. This is demonstrated in Steve’s words: ‘Well, I think growing my confidence, self-confidence, has helped. And I think it engaged me with a group of like-minded folk who became a tribe, as it were. And that gave me purpose.’

To summarize, gaining self-efficacy by overcoming challenging situations and testing one’s abilities is a transformational process. This was a prominent theme in respondents’ descriptions, and it positively impacted every aspect of their lives. Most agreed that improved self-efficacy and its associated benefits would not be apparent if they were not regularly active outdoors.

### 4.4. Challenge, Risk, and Coping

When individuals take part in adventure activities, they often deliberately seek out challenging situations and environments, benefiting considerably from testing themselves therein (Ewert & Davidson, 2021). Most respondents believed that a degree of self-challenge, a core psychological need (Huang et al., 2024), was integral to adventure, aligning with extant research (Buckley & Westaway, 2020). Despite this, there were mixed views about how challenge impacts well-being. For some respondents, pushing themselves out of their comfort zone was critical to their personal development and subsequent well-being. This is highlighted by Thomas, who commented that ‘if you don’t put yourself in a situation that you can’t completely control, you won’t get any further’ and ‘if you spend a lot of time outdoors, you will inevitably find yourself in borderline situations.’ Relatedly, Susan noted that ‘human bodies are designed for way more of this [pushing oneself]. It’s the mind we need to capture.’ Such comments point toward the inevitability of responding to challenges during activity participation. When “successful” in dealing with these demanding situations, respondents enjoyed positive affect and a boost to their HWB in the short term. Furthermore, the memories of these challenging times stayed with individuals, often becoming the key focus of their adventure narratives and leading to longer term EWB (Pomfret & Varley, 2019). Klara’s comments reflect these points:

‘There are probably thousands of examples where outdoor situations have helped me become more confident and resilient … giving me the trust to handle challenging situations. They’ve contributed to my development in a direction I value and enabled me to do many things … It’s comforting to know that I have that within me, that I could go out now and do something amazing. I find that incredibly valuable, and it’s become an important part of my life that I wouldn’t want to miss.’

These findings palpably demonstrate the transformational power of adventure experiences and how activity engagement develops good coping skills to deal with difficult situations in daily life. They align with the need for *self-challenge and competence* (Huang et al., 2024) and the key catalyzer of *risk, challenge and novelty* (Rus et al., 2022), supporting the view that challenges are essential for long-term transformation.

We acknowledge, however, that the challenging and risky elements of adventure can sometimes lead to strong negative affect and low self-efficacy. These are integral elements of EWB (Huta & Waterman, 2014) that can deter individuals from activity participation. For instance, Karsten recounted a time when they stopped enjoying adventure activities when training for a mountain marathon. They felt they were pushing themselves to the limit, commenting ‘that was also the first time I cancelled a marathon. I was completely overwhelmed and no longer enjoyed it. And then I ran into an injury … And a little later I started doing sport again and I realised that it wasn’t worth it if it wasn’t fun.’ In more extreme cases, a few respondents found themselves in situations where they felt they might get seriously injured or die, as exemplified in the comments from Steve:

‘And I have nearly died a few times, and I don’t say that in an overly inflated way. I have nearly died mountaineering a couple of times and when paragliding, I’ve nearly died once as well. So, yeah, they wake you up those experiences, although you never focus quite like it if you know what I mean. You know if you don’t perform here, you’re dead.’

Therefore, it is important for individuals to know their limits and to exert some degree of control over potential risks so as to benefit fully from their adventure activity experiences. Often, the build up to activity participation can trigger a heightened sense of perceived risk and fear. Once immersed in the activity, the experience can become less feared as reality takes over and people channel their energy into the “doing” part of the activity. Goran’s explanation of a scary adventure experience illustrates this:

‘If the fear is overwhelming you either give up, or you block it or freeze and then overcome the fear. For example, imagine falling into these glaciers. … While travelling to the Alps, the whole time I was thinking about it [falling in]. I felt uncomfortable about the trip, but when we eventually arrived and I found myself in front of the glacier, I realized how beautiful, in fact, it is to be there, and that there is nothing dangerous. It is just our own imagination. If you respect nature, there can’t be bad outcomes, so just by approaching and overcoming fears, they just go away.’

These findings emphasize the role of psychological needs and catalyzers in beneficial outdoor activity engagement. Emotions, peak experiences, and self-reflection are also important elements. It is evident that dealing with challenges could be emotional and unbalancing for a while, but self-reflection and enjoying a state of HWB from successfully overcoming the demanding situation catalyzed the transformation process for respondents.

## 5. Conclusions

This study makes its contribution by exploring the transformative impacts of nature-based adventure activity engagement and its long-lasting effects on eudaimonic well-being (EWB), specifically mental health. The findings palpably demonstrate that regular participation in adventure activities can transform individuals and encourage them to adopt an adventurous, active, and nature-oriented lifestyle with good levels of sustained EWB. Although there is plentiful research that confirms the mental health benefits of outdoor activity participation, as outlined in the Introduction (e.g., Buckley & Westaway, 2020; Davidson & Ewert, 2024; White et al., 2013), what sets our study apart from others is the focus on long-lasting transformation through adventure. We attempt to add to extant knowledge and connect transformation research to adventure contexts and enduring EWB. This is a scarcely researched area, yet it is a topic of importance given the ever present focus on well-being and good mental and physical health in society. Our work, therefore, advances the understanding of these themes and illustrates the differing and highly beneficial experiences that adventure engagement had on respondents. It acknowledges that adventure pursuit experiences are unique to the individual and affected by factors such as their emotional state, level of experience, and attitude toward the activity. This section outlines the theoretical and practical implications of the study’s findings, limitations, and future research directions.

### 5.1. Theoretical Implications

We now consider the theoretical implications of this study in the context of the three theories presented in Table 1. Our findings, which are summarized in Table 3, demonstrate that regular, long-term adventure activity engagement positively steers respondents toward psychological need fulfilment, as indicated in SDT (Deci & Ryan, 1985). Their narratives highlight the accomplishment of *autonomy, relatedness, and competence needs* through adventure, which aligns with previous studies (e.g., Houge Mackenzie & Hodge, 2020; MacGregor et al., 2014) but additionally confirms the importance of regular engagement over lengthier periods of time to sustain EWB and good mental health. Similarly, Huang et al.’s (2024) extended SDT framework of six psychological needs is also reflected in respondents’ comments. In fulfiling these needs, they strengthened their own skills, further developing these by seeking opportunities to experience liminality yet concurrently dealing with challenging natural environments so that they could continually test themselves and progress their abilities. Respondents enjoyed calming and relaxing natural settings where they could cherish special and transformative moments. A sense of “belonging” to nature and being part of a community of outdoor adventure enthusiasts were often mentioned in respondents’ recollections. Their stories also demonstrated how they thrived through beneficence, for instance, when introducing an adventure activity to others, or when doing something good for nature.

Rus et al.’s (2022) five key catalyzers of transformative tourism are also palpably reflected within this study’s findings. Respondents consistently alluded to challenge and sometimes risk as essential aspects of their adventure activity experiences, reflecting extant research (Buckley & Westaway, 2020; Ewert & Davidson, 2021). They talked about the value of engaging in authentic nature-based experiences in line with Pung and Del Chiappa’s (2020) work on existential authenticity. They appreciated the outdoors as a liminal place, which offered escapism from their daily lives, where they could be themselves and forget about their worries (Turner, 1974). Feeling connected to others and being part of a group, but also being part of this world, were integral aspects of respondents’ experiences. They emphasized this as a critical element of their transformation, reflecting identity development, as highlighted in Ashworth’s (2017) study. Pastor and Kent (2020) examined the importance of *emotions and peak experience* as transformation catalyzers. Our findings confirm these as essential elements gained during adventure activity engagement, leading to enhanced EWB. Furthermore, respondents’ adventure experiences were often vivid, memorable, and intense, supporting extant research that highlights the “quantum change” individuals go through during peak experiences (Naor & Mayseless, 2020). *Self-reflection* also acted as an important catalyzer (Pung & Del Chiappa, 2020), which was demonstrated in respondents’ observations about what they learned from their adventure experiences and how they transferred this to their daily lives. These findings support the power of outdoor adventure experiences to transform individuals, as highlighted by Arnould and Price (1993), and they might be “forever changed.”

Notably, this study’s findings highlight a range of different but intertwined themes that contribute to long-term EWB and good mental health. These span the themes presented within the SWB conceptual framework (Pomfret et al., 2023), SDT (Deci & Ryan, 1985), its variations (Houge Mackenzie & Hodge, 2020; Huang et al., 2024), and the transformational catalyzers (Rus et al., 2022). The experienced outdoor enthusiasts in this study continually benefited from regular activity engagement, thriving from the EWB benefits, including liminality, immersion in nature, self-efficacy, belonging to a community, and identity development. However, overcoming challenges, building up resilience, and developing coping strategies seem to impact long-term EWB most strongly. This is particularly pertinent whereby respondents transferred their skills to their everyday lives. Over the years, adventure activities were essential in maintaining respondents’ well-being, reducing ill-being and dealing with the constant demands incurred in life. They often expressed that they would feel as if something was missing from their lives if their activity participation was constrained in any way.

### 5.2. Practical Implications

This study’s findings can help organizations in the adventure industry to design adventure activity experiences that embed opportunities to appreciate both hedonic and eudaimonic states of well-being. This will not only enhance the positive perception of adventure products for individuals but will also encourage them to continually engage in their chosen adventure pursuits, either through commercially organized activities or independently. This will contribute to growth within the adventure industry through future bookings, repeat business, and consumer loyalty. The wide-ranging well-being and mental health benefits that regular adventure activity engagement offer, particularly for long-lasting EWB and transformation, are currently insufficiently marketed. Accordingly, our findings can assist with the promotion of these beneficial outcomes to potential consumers.

The practical implications of this research are not limited to the adventure industry and can also be applied to the health sector, specifically clinical psychology, and potentially influence public policy. Our findings could assist health officials with the design of adventure programs and interventions, such as adventure therapy and green social prescribing programs, to improve health and promote long-lasting EWB. These findings also offer insights into how to prevent the onset of mental health conditions and enhance mental health for those with diagnosed conditions through actively engaging with nature-based settings.

### 5.3. Limitations and Future Research

This research does not come without limitations. Firstly, the selection of the sample may have missed certain types of outdoor adventure enthusiast. Yet, we tried to ensure a broad range of respondents through our sampling strategy, which included respondents from three countries. Nonetheless, the findings cannot be generalized given that our respondents were European, which limits the applicability of this study’s findings to other cultural groups. Respondents recounted many positive adventure activity experiences over a long time, yet they concurrently endured some negative experiences, which affected their mental health. These negative elements can result in ill-being and prevent people from continuing to engage in their outdoor activities. However, negative experiences can often be integral to long-term EWB (Huta & Waterman, 2014), so long as feelings of ill-being do not persist during or after activity engagement.

While evidence on the positive effects of adventure activities on physical and mental health is growing, there is still a lack of long-term data (Britton et al., 2020). Future research should address this, as well as focus on the catalyzers of transformation in an outdoor setting. It should also be more interdisciplinary, bringing together public health, recreation, and tourism perspectives. Further work could additionally investigate what prevents people from being active outdoors, identifying barriers and transformative strategies as to how they can be encouraged to start adventure activities.

## Figures and Tables

**Table 1:** Key theoretical concepts related to SWB, SDT, and transformative catalyzers.

Key SWB Metathemes and Subthemes	SDT	Extended SDT	Transformation Catalyzers
(Pomfret et al., 2023)	(Deci & Ryan, 1985; Houge Mackenzie & Hodge, 2020)	(Houge Mackenzie & Hodge, 2020; Huang et al., 2024)	(Rus et al., 2022)
*Physical and mental balance*: physical health, mental health and emotional balance, challenge and risk, coping and resilience		Self-challenge, competence	Risk, challenge, and novelty
*Immersion and transformation*: antistructural experiences/liminality, human-nature, rhythm of nature and resonance, deceleration and mindfulness		Aesthetics and relaxation, escapism, relatedness	Authenticity and liminality
*Extraordinary experiences*: natural highs, transcendental experiences and awe, optimal flow and peak experiences		Relatedness	Emotions and peak experiences
*Personal development*: learning and knowledge acquisition, flourishing, individual identity	Autonomy, competence	Relatedness, education	Self-reflection
*Community*: collective identity, beneficence	Relatedness, beneficence	Relatedness, beneficence	Social dynamics and communitas

**Table 2:** Respondents’ age categories.

Age Groups	Number of Respondents	Percentages
18–24	4	10%
25–34	5	12.5%
35–44	10	25%
45–54	11	27.5%
55–64	10	25%

**Table 3:** Findings corresponding to the key research themes.

Key Research Themes	Findings
*Physical and mental balance* (Pomfret et al., 2023); *self-challenge and competence* (Huang et al., 2024); *risk, challenge, and novelty* (Rus et al., 2022)	- Self-efficacy: Testing one’s abilities and gaining self-belief - Overcoming challenges - Pushing yourself out of your comfort zone but knowing your limits - Controlling your fears
*Immersion and transformation* (Pomfret et al., 2023); *aesthetics and relaxation, escapism, relatedness* (Huang et al., 2024); *authenticity and liminality* (Rus et al., 2022)	- Emotional balance through nature and activity - Liminality: A space without worries - Self-worth and identity
*Extraordinary experiences* (Pomfret et al., 2023); *relatedness* (Huang et al., 2024); *emotions and peak experiences* (Rus et al., 2022)	- Transformative power of nature - Connection to nature through authentic experiences - Fun, positive feelings and emotions
*Personal development* (Pomfret et al., 2023); *autonomy and competence* (Deci & Ryan, 1985; Houge Mackenzie & Hodge, 2020); *relatedness and education* (Huang et al., 2024); *self-reflection* (Rus et al., 2022)	- Learning outdoors and gaining knowledge - Making decisions - Mindfulness techniques and coping strategies - Self-worth - Feeling in control, feeling capable, and “feeling like me” - Identity building
*Community* (Pomfret et al., 2023); *relatedness and beneficence* (Deci & Ryan, 1985; Houge Mackenzie & Hodge, 2020; Huang et al., 2024); *social dynamics and communitas* (Rus et al., 2022)	- Being part of organizations and clubs - Participating in adventures with friends and family - Belonging to a community - Identity development

## Data Availability

Restrictions apply to the availability of this data. Interview data were stored as audio and transcript files on the research team’s university laptops. To ensure confidentiality for the interview respondents, the data were restricted to and only accessed by each individual researcher from their university laptops throughout the project. Interviewees were required to complete a participant consent form; these were also stored on the research team’s university laptops. Respondents’ identities were anonymized by allocating them a code and pseudonym name. Requests to access the data should be directed to the corresponding author: g.c.pomfret@shu.ac.uk.
